# ABCB1-Mediated Drug Efflux Drives Resistance to VpreB1-Targeted Antibody-Drug Conjugates in B-cell Lymphoblastic Leukemia

**DOI:** 10.21203/rs.3.rs-11166558/v1

**Published:** 2026-10-21

**Authors:** Robin L. Williams, Xiaohong Wang, Jason Ostergaard, Jongseok Kang, Madison Gohman, Luke Lambert, Timothy Singleton, Sarah K. Tasian, Megan Hilgers, Keegan C. Lee, Joseph M. Muretta, Stuart S. Winter, Peter M. Gordon

## Abstract

B-lineage acute lymphoblastic leukemia (B-ALL) is highly responsive to immunotherapy, yet resistance remains a major barrier to durable responses. We developed a VpreB1 (CD179a)-directed antibody-drug conjugate (VpreB1-ADC) that exploits the restricted expression of VpreB1 within the surrogate light chain in early B cells and B-ALL. This calicheamicin-based ADC induces potent cytotoxicity and durable remissions in preclinical models, although a subset of leukemia cells exhibits intrinsic resistance. Here, we investigated mechanisms of VpreB1-ADC resistance in a TCF3::HLF-rearranged B-ALL model. Resistant cells retained VpreB1 surface expression and efficient antibody internalization, excluding defective target engagement. Instead, resistant cells exhibited elevated expression and activity of the multidrug resistance transporter ABCB1 (P-glycoprotein). Pharmacologic inhibition of ABCB1 with tariquidar or zosuquidar restored VpreB1-ADC sensitivity. Conversely, enforced ABCB1 overexpression in multiple B-ALL cell lines conferred resistance to VpreB1-ADC, which was reversed by ABCB1 inhibition. Notably, leukemia cells with high ABCB1 activity remained sensitive to cytotoxic agents that are validated ADC payloads, including exatecan and PNU-159682, which are not ABCB1 substrates. These findings identify ABCB1-mediated drug efflux as a mechanism of resistance to VpreB1-ADC and support alternative payload selection to circumvent this resistance mechanism, with implications for optimizing ADC design and combination therapies.

